# Unravelling Acquired Hemophilia A in an Ambiguous Clinical Picture

**DOI:** 10.7759/cureus.68549

**Published:** 2024-09-03

**Authors:** Fathima Shehnaz Ayoobkhan, Dakshin S Padmanabhan, Rula Mahayni, Sara Riaz, Geetha Krishnamoorthy

**Affiliations:** 1 Internal Medicine, Trinity Health Oakland Hospital, Pontiac, USA; 2 Infectious Disease, Trinity Health Oakland Hospital, Pontiac, USA; 3 Hematology and Medical Oncology, Corewell Health Beaumont Troy Hospital, Troy, USA

**Keywords:** renal hemorrhage, cydri regimen, factor viii inhibitor assay, factor viii activity, acquired hemophilia a (aha)

## Abstract

Acquired hemophilia A (AHA) is characterized by the development of neutralizing autoantibodies, called "inhibitors," against intrinsic factor VIII. Its presentation differs profoundly from congenital hemophilia. Here, we present the case of a 69-year-old patient presenting with right-sided flank pain and hematuria, initially diagnosed with acute pyelonephritis, who was found to have bilateral renal hemorrhage during the course of his hospitalization. Later, after a thorough diagnostic evaluation, he was deemed to have AHA.

## Introduction

Acquired hemophilia A (AHA) is a rare catastrophic bleeding disorder caused by the development of neutralizing autoantibodies, called "inhibitors," against intrinsic factor VIII, with an incidence of 1.5 million cases a year [1,2]. Several risk factors, such as malignancy, pregnancy, and autoimmune disorders, including systemic lupus erythematosus (SLE) and rheumatoid arthritis (RA), are associated with the development of factor VIII inhibitors [3-5]. Unfortunately, almost 50% of patients have no associated risk factors, making this an elusive diagnosis that requires heightened awareness to identify [6]. AHA has an overall mortality rate ranging from 8% to 33%, emphasizing the importance of early diagnosis [7]. AHA should be suspected in patients presenting with active bleeding and an isolated prolongation of the activated partial thromboplastin time (aPTT). Factor VIII activity, along with factor VIII inhibitor (Bethesda) assay, establishes the diagnosis [8]. Unlike congenital hemophilia, AHA may not manifest the classical "hemarthrosis"; instead, other presentations such as soft tissue, muscle, or mucosal bleeding are common [9].

We describe a 69-year-old man presenting with right-sided flank pain and hematuria, initially diagnosed with acute pyelonephritis. Later, after a thorough diagnostic evaluation, he was deemed to have AHA. We aim to increase awareness of this diagnosis and the myriad presentations associated with AHA, benefiting hematologists as well as the general medical community.

## Case presentation

A 69-year-old man with a history of prostate cancer under clinical remission and chronic obstructive pulmonary disease (COPD) presented with sudden onset right flank pain, fever (101 °F), and hematuria for two days. Physical examination was notable for right lower quadrant tenderness to palpation. The clinical picture suggested right-sided pyelonephritis versus acute appendicitis. Laboratory results were significant for mild leukocytosis of 11,400/µL; urinalysis showed bacteriuria, and the patient was started on ceftriaxone. CT imaging demonstrated mild hydroureteronephrosis with inflammatory stranding of the right kidney, and a 7 mm appendicolith was noted. CT imaging was consistent with recently passed calculus versus ascending pyelonephritis. The patient’s clinical status improved, and he was discharged on oral cephalexin.

**Figure 1:**
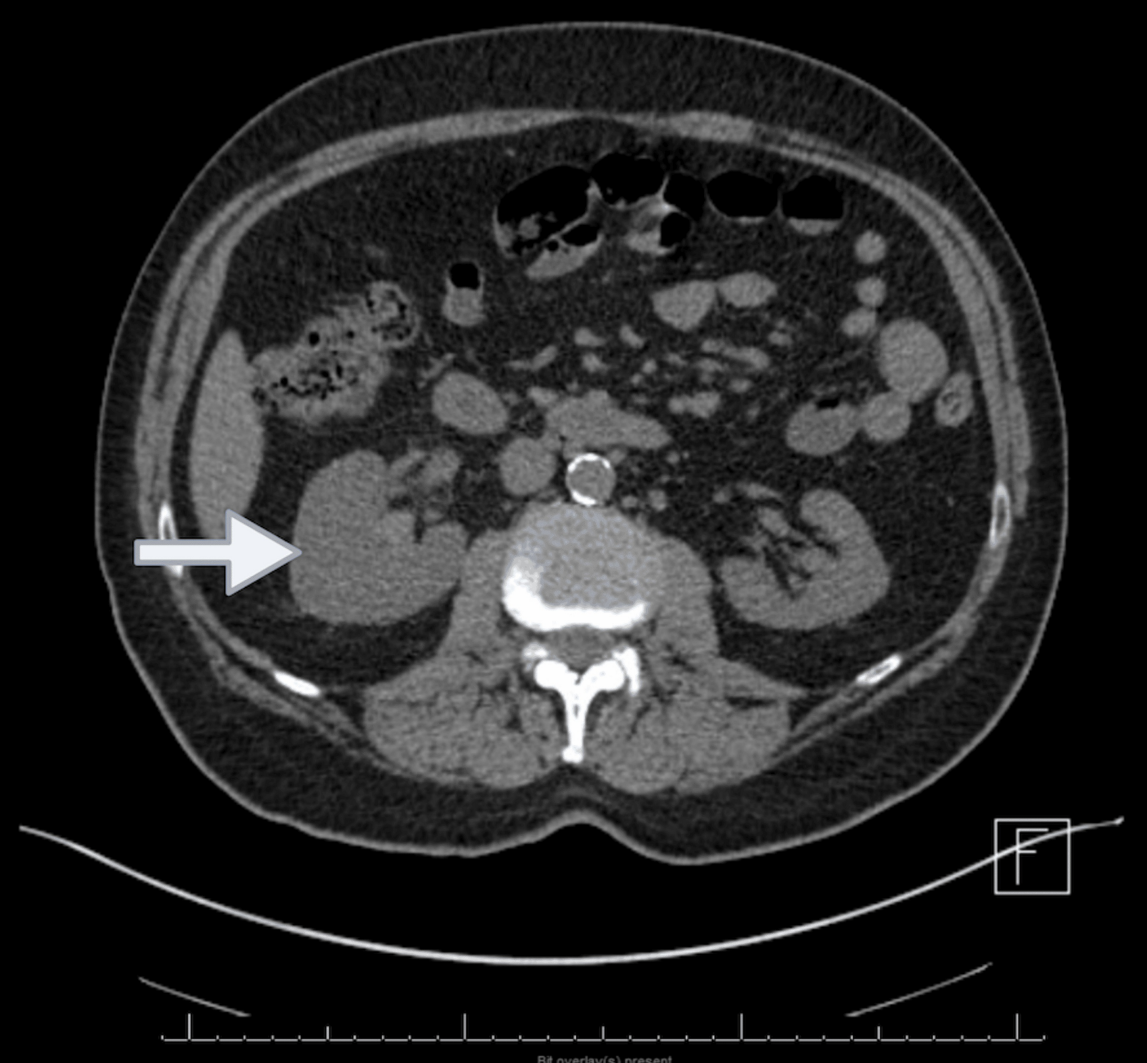
CT abdomen/pelvis showing initial right renal hemorrhage with right hydronephrosis (arrow)

Four days post-discharge, the patient presented again to the hospital with unresolved right flank pain, hematuria, and a fever of 102 °F. He was non-compliant with antibiotics at discharge. Suprapubic tenderness was noted. Labs revealed acute kidney injury (AKI): creatinine was 1.66 mg/dL (baseline creatinine 0.9-1 mg/dL). CT scan showed right hydroureteronephrosis and hemorrhage in the right renal collecting system (Figure 1). He was started on IV cefazolin for the possibility of partially treated pyelonephritis. Antibiotics were switched to oral cephalexin. However, the patient developed recurrent fever, and treatment was escalated to IV cefepime. Nephrology evaluated the etiology of AKI. IV cefepime 2 g every 12 hours for a 10-day duration was switched to cefpodoxime 200 mg PO every 12 hours to complete 14 days of therapy due to concern for cefepime-induced AKI. Right cystoscopy was performed along with right retrograde pyelogram, right ureteroscopy, stent placement over the right kidney, and biopsy of the right renal pelvis, which showed inflammatory changes without evidence of renal malignancy.

**Figure 2:**
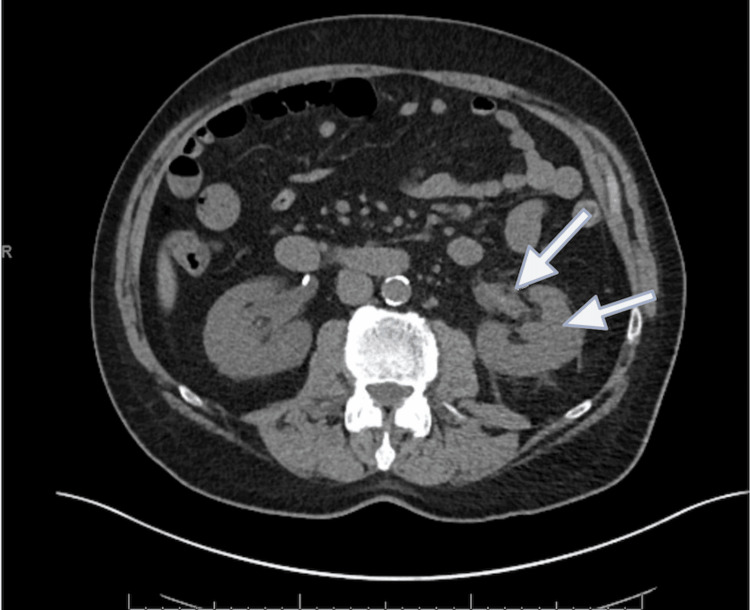
CT abdomen/pelvis on later presentation showing left renal hemorrhage and hydronephrosis (arrows)

Five days after the second discharge, the patient presented again with left-sided flank pain and hematuria, with the passage of clots. He was noted to have anemia (Hb 9.2) and a worsening renal profile (creatinine 7 mg/dL). CT scan revealed blood within the left renal collecting system and proximal ureter, associated with evidence of pyelonephritis, and fullness was also noted in the right renal collecting system with a patent nephroureteral stent (Figure 2). The patient was started on IV doxycycline and ciprofloxacin for the treatment of pyelonephritis due to concern for allergic interstitial nephritis.

Investigation

**Figure 3:**
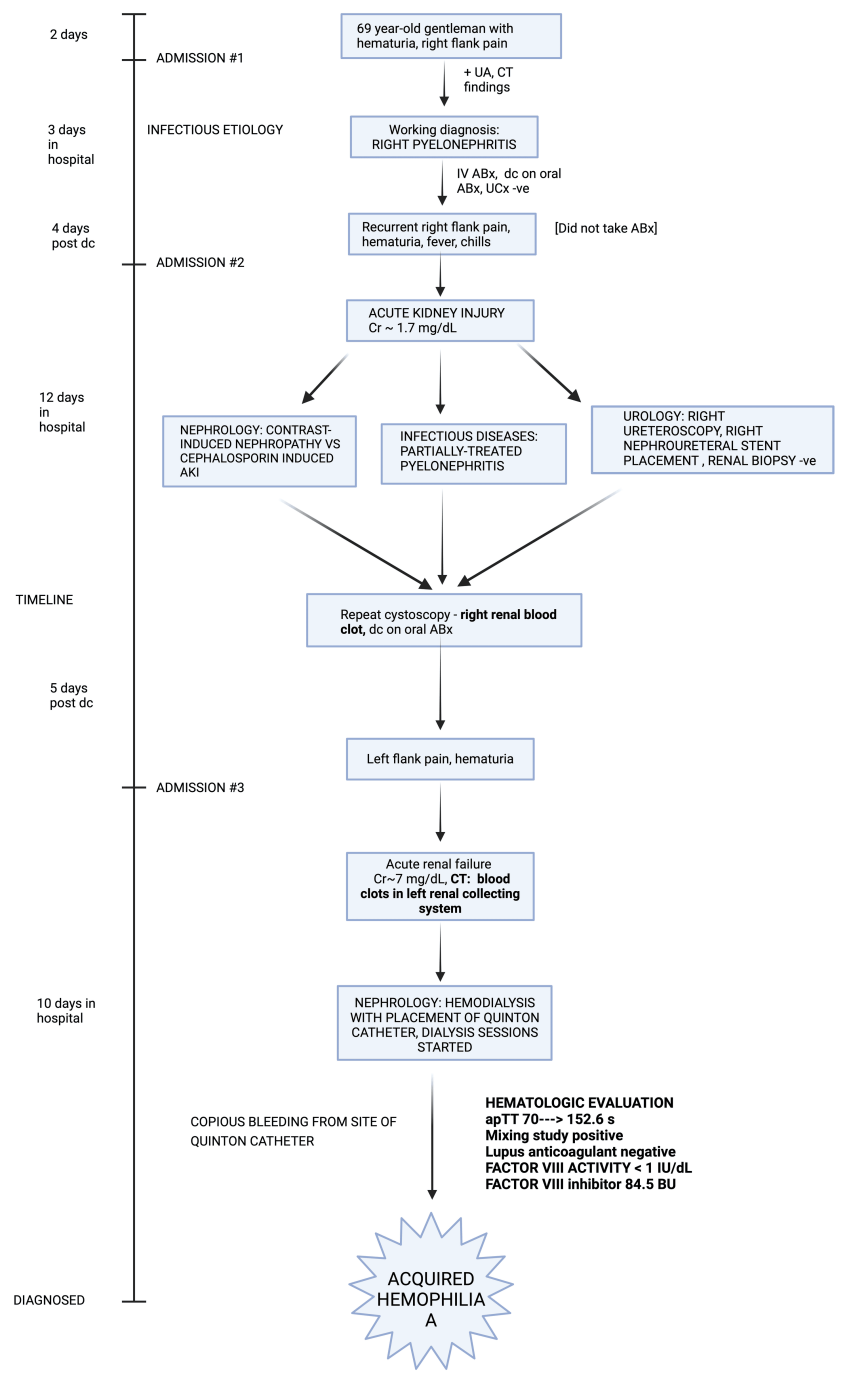
Sequence of events that led to the final diagnosis of acquired hemophilia A Image created with BioRender software.

Four cycles of hemodialysis were performed successfully; however, bleeding was noticed from the site of the hemodialysis catheter. This prompted an initial coagulation panel, which showed an elevated aPTT as high as 152.6 s (reference values 25.1-38.5 s). Further investigations revealed that lupus anticoagulant was negative, the mixing study suggested the presence of an inhibitor as aPTT failed to correct, Factor VIII activity was less than 1 IU/dL, and the Factor VIII (Bethesda) inhibitor assay titer was profoundly elevated at 84.5 Bethesda Units (BU). The final diagnosis was consistent with AHA (Figure 3).  

Treatment

The treatment involved receiving 4 units of fresh frozen plasma (FFP) transfusion for prolonged aPTT, with no resolution. Recombinant factor VIIa was administered for 2 doses, and daily infusions of factor VIII concentrate were given for 10 days. Management per the treatment algorithm to eradicate the inhibitor included IV Rituximab 375 mg/m² for 4 weekly doses and Prednisone 60 mg PO daily.

Outcome

Treatment with Rituximab improved hematuria, and factor VIII activity increased to 4 IU/dL. After eight sessions of dialysis, renal status normalized. He completed four cycles of Rituximab and continued on Prednisone 60 mg throughout the treatment. A PET scan was unremarkable for malignancy, with a normal PSA level of 1.2 ng/mL. After completing four cycles of Rituximab, he followed up with his hematologist with complaints of epistaxis and hematuria. Labs were significant for an elevated aPTT of 55.6 s, factor VIII activity increased to 12 IU/dL but was still not within the normal range, and factor VIII inhibitor had notably reduced to 2 BU but was still persistent. Despite some improvement, and given that the patient had bleeding symptoms, his treatment plan was escalated to cyclophosphamide, four additional cycles of Rituximab, and weekly dexamethasone, or "CyDRi" regimen [10]. As of this writing, the patient completed two cycles of this regimen with a good response, with factor VIII activity now within the normal range at 68 IU/dL. Factor VIII inhibitor levels are pending. aPTT is also within the normal range at 37.3 s.

## Discussion

Our case was unique in that the initial causes of bleeding with hematuria and renal hemorrhage were thought to be associated with infectious, urological/obstructive, and even intrinsic renal pathologies such as contrast-induced nephropathy and allergic interstitial nephritis secondary to cephalosporin antibiotics. Upon reviewing the coagulation profile after bleeding from the dialysis catheter site, aPTT was elevated, which prompted further hematological work-up and the final diagnosis of AHA. In medical practice, it is always a tried and tested approach to think of common causes of clinical presentations first, so the initial differential diagnoses were all equally plausible. The systematic multi-departmental approach that was taken to find this rare diagnosis further adds to the intriguing nature of this case.

Ajjikuttira et al. reported a case of AHA presenting with left lower abdominal pain and hematuria initially, with CT imaging revealing a blood clot within the left renal tract. The patient also had AKI. On initial presentation, aPTT was elevated at 50 s. The working diagnosis was hemorrhagic pyelitis, and the patient was treated with IV ceftriaxone and discharged once AKI and hematuria resolved. He presented again with contralateral right-sided flank pain and hematuria. Repeat CT showed blood products in the right renal pelvis and ureter. At this time, the patient also had nephrotic range proteinuria; however, glomerulonephropathy screen was negative. Further work-up performed by Hematology revealed positive lupus anticoagulant, low factor VIII activity at less than 1 IU/dL, and positive factor VIII inhibitor at 12.5 BU, supporting the diagnosis of progressing AHA masked by positive lupus anticoagulant [11]. The patient’s treatment regimen differed in that he was treated with factor VIII inhibitor bypass activity (FEIBA) and cyclophosphamide with prednisolone for factor VIII inhibitor eradication. This case is perhaps similar to our patient in that infectious and renal pathologies were considered first, and then hematological etiologies were revealed. Another case reported by Otaki et al. describes a 53-year-old woman presenting with acute renal failure and macrohematuria, with CT showing bilateral kidney swelling and dilatation of the renal pelvis. Further hematologic work-up showed factor VIII activity low at 1.8 IU/dL and factor VIII inhibitor elevated at 19 BU [12]. This patient was treated with hemodialysis, cyclophosphamide, and prednisolone, but no bypassing agent was specifically used. Rituximab as the initial immunosuppressive medication was not used in either case.

The key to successful AHA treatment is prompt diagnosis and tailored treatment. Managing AHA involves controlling active bleeding, preventing further episodes of bleeding, and neutralizing factor VIII inhibitors, in this specific order of importance [2].

The efficacy of treatment is tracked through close clinical monitoring of the patient for signs of bleeding. It should be noted that despite the importance of factor VIII inhibitor levels and factor VIII activity levels for making the diagnosis of AHA, their exact numerical values do not dictate when to initiate hemostatic treatment in patients who have clinically relevant bleeding [13]. These initial agents include FEIBA and recombinant factor VIIa, of which the latter was used in this case. There is no clear benefit of one of these drugs over the other [14,15]. These medications are referred to as “bypassing agents”, treatments that can activate the coagulation cascade independently of factors VIII and IX and hence are not affected by the presence of factor VIII or IX inhibitors. In the EACH2 trial conducted by Baudo et al., delay in initiation of treatment remained a significant factor in patient response to treatment [14].

Emicizumab, a bispecific factor VIII mimetic therapeutic antibody used in managing congenital hemophiliacs, has not been well-studied in AHA. Knoebl et al. performed a study in which 12 subjects were treated with emicizumab as hemostatic therapy for AHA. Initial therapy involving standard-of-care bypassing agents (recombinant factor VIIa or FEIBA) was followed by emicizumab administration weekly. It resulted in the achievement of good hemostatic efficacy in these patients. It was observed that within a few days after the first injection of emicizumab, less bypassing therapy was required to achieve hemostasis [16]. A dedicated clinical trial for emicizumab in AHA is essential for its consideration in the treatment regimen as standard-of-care in the future.

The pursuit to eradicate the inhibitor is facilitated by the use of immunosuppressive therapies, which involve corticosteroids, cyclophosphamide, high-dose immunoglobulins, and now perhaps the most effective agent of them all, rituximab, with a response rate reported as high as 90% [17,18]. A combination of corticosteroids at a dose of 1 mg/kg with rituximab at a dose of 375 mg/m² to a maximum of four doses, or any other cytotoxic agent for first-line therapy, may be beneficial in patients with FVIII < 1 IU/dL or inhibitor titer > 20 BU [13]. In our case, the patient was given a treatment regimen of prednisone and rituximab, which significantly helped with hematuria.

Prognostication of treatment response involves re-checking factor VIII activity and factor VIII inhibitor levels, with partial remission (PR) traditionally being described as factor VIII activity being restored to a level > 50 IU/dL and complete remission (CR) being defined as PR plus a negative, completely undetectable factor VIII inhibitor level. A pivotal study in AHA prognostication performed by Tiede et al. concluded that a baseline factor VIII activity < 1 IU/dL and factor VIII inhibitor level > 20 BU at initial diagnosis was associated with decreased survival [19]. Our patient had poor prognostic indicators as mentioned above on both counts and required further treatment with the "CyDRi" regimen, where he was noted to have a PR with two of the four total cycles, with factor VIII activity levels returning to the normal range.

Given the rarity of this condition, clinicians have trouble reaching this subtle clinical diagnosis. A survey-based study among physicians across multiple clinical specialties conducted by Reding et al. reported that lack of appropriate attention to bleeding and prolonged aPTT was a significant barrier to the diagnosis and treatment of AHA, and that hematology consultation was a potentially effective strategy to break down this barrier [20]. Our case epitomizes the results of the above study rather emphatically.

## Conclusions

Our clinical vignette describes the ambiguity in the presentation of AHA and the difficulty in reaching the final diagnosis. Heightened awareness in a patient with bleeding and prolonged aPTT is paramount to involving expert hematological evaluation and management strategies early to achieve meaningful clinical remission in this potentially fatal bleeding disorder.

## References

[1] Kruse-Jarres R, Kempton CL, Baudo F (2017). Acquired hemophilia A: updated review of evidence and treatment guidance. Am J Hematol.

[2] Franchini M, Vaglio S, Marano G, Mengoli C, Gentili S, Pupella S, Liumbruno GM (2017). Acquired hemophilia A: a review of recent data and new therapeutic options. Hematology.

[3] Hauser I, Lechner K (1999). Solid tumors and factor VIII antibodies. Thromb Haemost.

[4] Tengborn L, Baudo F, Huth-Kühne A (2012). Pregnancy-associated acquired haemophilia A: results from the European Acquired Haemophilia (EACH2) registry. BJOG.

[5] Soriano RM, Matthews JM, Guerado-Parra E (1987). Acquired haemophilia and rheumatoid arthritis. Br J Rheumatol.

[6] Franchini M, Gandini G, Di Paolantonio T, Mariani G (2005). Acquired hemophilia A: a concise review. Am J Hematol.

[7] Mulliez SM, Vantilborgh A, Devreese KM (2014). Acquired hemophilia: a case report and review of the literature. Int J Lab Hematol.

[8] Kasper CK, Aledort L, Aronson D (1975). Proceedings: A more uniform measurement of factor VIII inhibitors. Thromb Diath Haemorrh.

[9] Knoebl P, Marco P, Baudo F (2012). Demographic and clinical data in acquired hemophilia A: results from the European Acquired Haemophilia Registry (EACH2). J Thromb Haemost.

[10] Simon B, Ceglédi A, Dolgos J (2022). Combined immunosuppression for acquired hemophilia A: CyDRi is a highly effective low-toxicity regimen. Blood.

[11] Ajjikuttira A, Sharma P, Rhee H (2020). Bilateral renal haematuria and obstructive renal failure from acquired haemophilia A: a medical cause for a surgical problem. BMJ Case Rep.

[12] Otaki Y, Kouda R, Fujimura T (2010). Acute renal failure as a complication of acquired hemophilia due to autoantibody to factor VIII. Clin Exp Nephrol.

[13] Tiede A, Collins P, Knoebl P (2020). International recommendations on the diagnosis and treatment of acquired hemophilia A. Haematologica.

[14] Baudo F, Collins P, Huth-Kühne A (2012). Management of bleeding in acquired hemophilia A: results from the European Acquired Haemophilia (EACH2) Registry. Blood.

[15] Tiede A, Worster A (2018). Lessons from a systematic literature review of the effectiveness of recombinant factor VIIa in acquired haemophilia. Ann Hematol.

[16] Knoebl P, Thaler J, Jilma P, Quehenberger P, Gleixner K, Sperr WR (2021). Emicizumab for the treatment of acquired hemophilia A. Blood.

[17] Collins PW (2007). Treatment of acquired hemophilia A. J Thromb Haemost.

[18] Franchini M, Veneri D, Lippi G, Stenner R (2006). The efficacy of rituximab in the treatment of inhibitor-associated hemostatic disorders. Thromb Haemost.

[19] Tiede A, Klamroth R, Scharf RE (2015). Prognostic factors for remission of and survival in acquired hemophilia A (AHA): results from the GTH-AH 01/2010 study. Blood.

[20] Reding MT, Cooper DL (2012). Barriers to effective diagnosis and management of a bleeding patient with undiagnosed bleeding disorder across multiple specialties: results of a quantitative case-based survey. J Multidiscip Healthc.

